# Preparation of Complex Glycans From Natural Sources for Functional Study

**DOI:** 10.3389/fchem.2020.00508

**Published:** 2020-07-03

**Authors:** Qing Zhang, Zhonghua Li, Xuezheng Song

**Affiliations:** Department of Biochemistry, Emory Comprehensive Glycomics Core, Emory University School of Medicine, Atlanta, GA, United States

**Keywords:** natural glycans, CORA, oxidative release, HPLC, tagging, large scale preparation

## Abstract

One major barrier in glycoscience is the lack of diverse and biomedically relevant complex glycans in sufficient quantities for functional study. Complex glycans from natural sources serve as an important source of these glycans and an alternative to challenging chemoenzymatic synthesis. This review discusses preparation of complex glycans from several classes of glycoconjugates using both enzymatic and chemical release approaches. Novel technologies have been developed to advance the large-scale preparation of complex glycans from natural sources. We also highlight recent approaches and methods developed in functional and fluorescent tagging and high-performance liquid chromatography (HPLC) isolation of released glycans.

## Introduction

Glycans, as one of the four major biological macromolecules in mammalian systems, are the most diverse and abundant biopolymers (Ohtsubo and Marth, 2006). Besides serving as structural support (such as cellulose) and energy storage (such as starch and glycogen), many glycans are covalently linked to proteins or lipids and play a wide variety of functional roles in physiological and pathophysiological states (Varki, 2017; Reily et al., 2019). Aberrations of glycan structures are associated with many diseases, including cancer, autoimmune, infectious, chronic inflammatory diseases, etc. (Reily et al., 2019).

Recently, glycoscience and functional glycomics have greatly advanced to systematically study the structure and function of glycans (Paulson et al., 2006; Cummings, 2009; Taniguchi et al., 2009; Smith and Cummings, 2013; Cummings and Pierce, 2014; Song et al., 2015). However, the functional study of glycans and glycoconjugates lags far behind those of proteins/peptides and nucleic acids. This is partially due to the fact that glycosylation is a post-translational modification, and the biosynthesis of glycans is not directly template-driven. Glycans are often highly branched structures as products of concerted reactions by glycosyltransferases and/or glycosidases. As a result, both high-throughput structural characterization (sequencing) and automated synthesis/expression are yet in the infant stage. Nevertheless, the importance of biological functions of glycans has been more and more recognized, driving significant interests to glycoscience study. Over the last decades, many new methods and technologies, such as those based on high-performance liquid chromatography (HPLC), mass spectrometry (MS), and LC-MS, have been developed to facilitate glycoscience study (Royle et al., 2008; Zaia, 2008; Doneanu et al., 2009; Ruhaak et al., 2010). Among those, the glycan microarray has proved to be very successful as a high-throughput screening tool for protein–glycan interactions. A glycan microarray is a presentation of a library of diverse glycan structures on a solid surface, such as microscope glass slides, for interrogation with fluorescently tagged glycan binding proteins (GBPs). As the biological functions of glycans are often realized through their specific interaction with GBPs, the glycan microarray has become extremely useful in elucidating ligand specificity of GBPs and generating biological hypothesis based on protein–glycan interactions (Fukui et al., 2002; Stowell et al., 2010; Song et al., 2014a, 2015; Smith et al., 2019). For a glycan microarray to be useful, the expansion of glycan libraries with more diverse and biomedically relevant structures is critical for advancing functional glycomics (Song et al., 2014a). The lack of more of these glycan structures for structural and functional study is a general problem for nearly all aspects of glycoscience. To address this problem, currently there are two main approaches to prepare glycans: chemical/chemo-enzymatic synthesis and isolation/separation of glycans from natural sources. Chemo-enzymatic approaches have been developed for the synthesis of structurally defined glycans in the last two decades (Koeller et al., 2000; Blixt and Razi, 2006; Boltje et al., 2009; Lepenies et al., 2010; Palcic, 2011; Schmaltz et al., 2011). A lot of effort and various synthetic methods have been introduced to make more complex glycans available (Wang et al., 2013, 2018; Chen, 2015; Li et al., 2015; Shivatare et al., 2016; Prudden et al., 2017; Zhang et al., 2017; Wen et al., 2018; Liu et al., 2019), and recently, two enzyme-mediated oligosaccharide synthesizers were reported to facilitate the synthetic progress (Zhang et al., 2018; Li et al., 2019). Despite many recent advancements in prototypic automated glycan synthesis, the synthesis of complex, highly branched glycan structures is still extremely challenging and can only be carried out in a number of noncommercialized laboratories. In addition, chemical/chemoenzymatic synthesis is target-driven, and the selection of biomedically relevant structures as synthetic targets relies on preliminary structural and functional analysis of natural glycome (Song et al., 2014a). On the other hand, the preparation of natural glycans has been traditionally carried out at μg scales for structural analysis. Because the biomedically relevant glycan structures often exist at low abundance and as heterogeneous glycoconjugates, the challenges to isolate sufficient quantities in high purity and define their structures are also high. Nevertheless, due to their higher potential biomedical relevance and lower technical barrier to access, we consider the production of natural glycan preparation for functional study to be an important and indispensable approach for glycoscience and functional glycomics.

**Figure 1:**
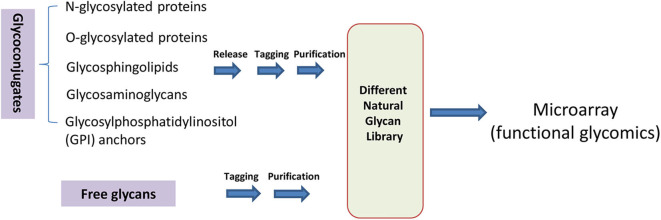
Preparation of natural glycans for functional glycomics.

In general, natural glycans occur in two categories: covalently attached to other biomolecules as glycoconjugates and free reducing glycans existing in organisms. The preparation of glycans from glycoconjugates requires the release of glycans first. Then glycans can be tagged, purified, and separated based on their physical and chemical properties. In this review, we discuss the diverse approaches for preparing different classes of nature glycans, including N-glycans, O-glycans, glycosphingolipids, glycosaminoglycans, glycosylphosphatidylinositol (GPI)-anchor glycans, and human milk oligosaccharides (HMOs). Glycans released from diverse natural glycoconjugates on cells or free glycans can be extracted, tagged, and purified to expand natural glycan libraries. These natural glycans can be printed onto glass slides as microarrays for functional glycomics study (Figure 1).

## N-Glycan Release From Natural Glycoproteins

**Figure 2:**
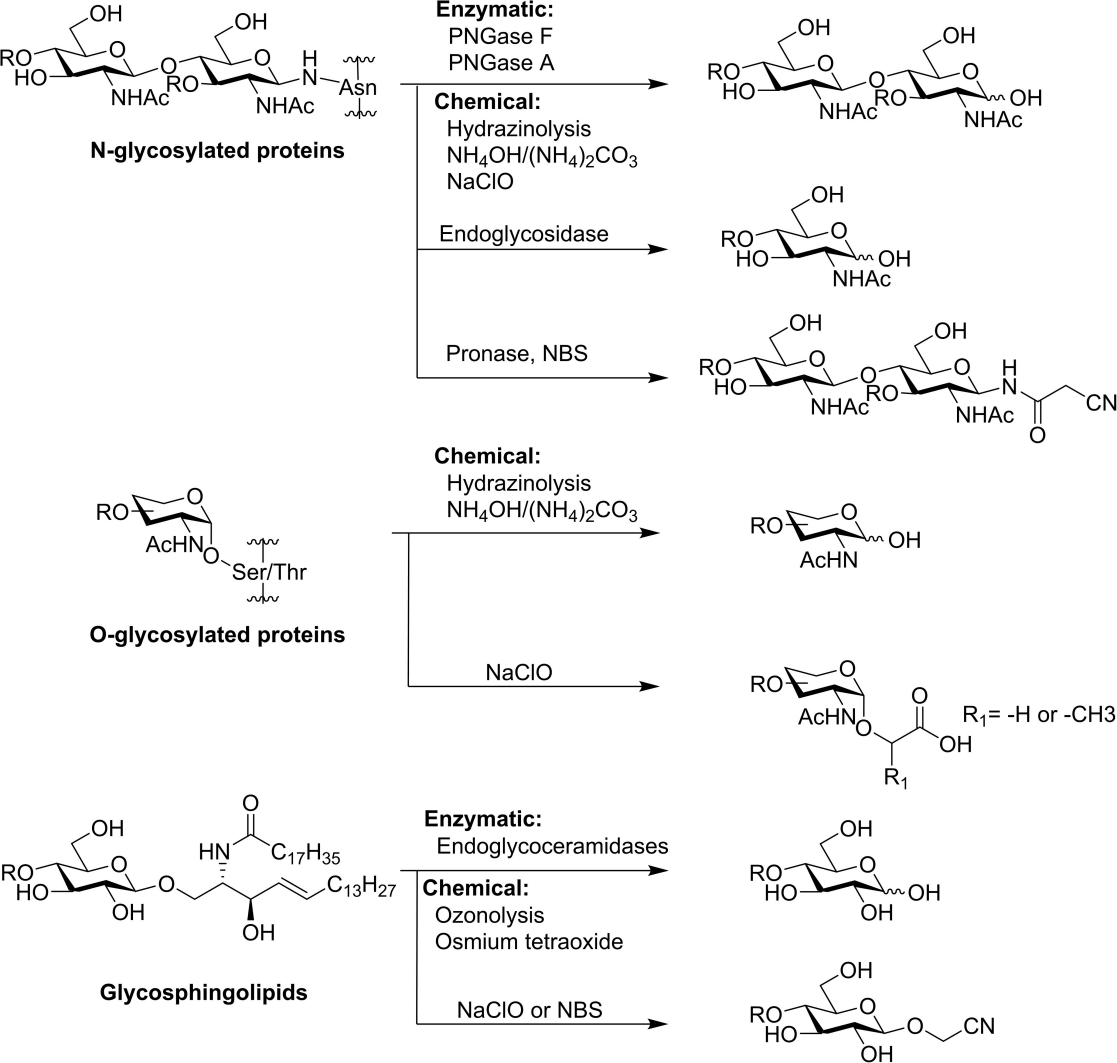
Common method to release glycans from glycoproteins and glycosphingolipids.

As the most well-studied class of glycans until now, N-glycans can be cleaved off glycoproteins by several enzymes, such as Peptide-N-Glycosidases (PNGase) and endoglycosidases (Endo) (Figure 2). PNGase F is the most widely used enzyme to remove *N*-glycans from most *N*-linked glycoproteins and glycopeptides except core α3-fucosylated N-glycans, which are commonly found in plants and insects (Plummer et al., 1984; Tarentino et al., 1985; Tretter et al., 1991). PNGase A has broader substrate specificity and can cleave core α1–3-fucosylated N-glycan (Takahashi, 1977). Recently, PNGase F-II, acid-stable PNGase H^+^, and PNGase Yl from yeast are all reported to release core α3-fucosylated N-glycans (Du et al., 2015; Lee et al., 2015; Sun et al., 2015). PNGase Ar is able to release the unusual GalαFucα1,3-reducing terminal core from *Caenorhabditis elegans* (Yan et al., 2018). As another option for enzymatic *N*-glycan release, endoglycosidases are able to cleave the β1–4-linkage of the di-*N*-acetylchitobiose core, and such enzymes include Endo A, Endo H, Endo M, Endo D, and Endo S (Freeze and Kranz, 2008; Huang et al., 2012; Wang and Amin, 2014; Li et al., 2016). Although they cleave *N*-glycans at the same position, they have different substrate specificities related to the structures of the *N*-glycans (Fairbanks, 2017). Although it is not a focus of this review, it is worth noting that many mutants of endoglycosidases have been developed as synthases for N-glycopeptides and glycoproteins (Huang et al., 2012; Wang and Amin, 2014). The high cost of PNGases and endoglycosidases limits their application in large-scale preparation of N-glycans. Another enzymatic approach is using pronase to cleave peptide bonds and leave glycan-peptide linkages intact (Dodds et al., 2009; Song et al., 2009a; Lu et al., 2019). Pronase is much cheaper than PNGases and endoglycosidases, but its full digestion of glycoproteins to glycoamino acids is always a challenge and often difficult to reproduce.

Chemical release approaches have provided an alternative to solve the high cost of enzymatic approaches in large preparation of N-glycans. Hydrazinolysis and ammonia/ammonium carbonate have been shown to release N-glycans from glycoproteins (Yosizawa et al., 1966; Huang et al., 2001; Nakakita et al., 2007). However, toxic reagents and/or harsh conditions are necessary, which is not amenable to large-scale preparation and may seriously affect the structural integrity of the released glycans. Under a set of optimized milder alkaline conditions, N-glycans without core α1–3-fucose can also be released by selective hydrolysis of N-glycopeptide (Yuan et al., 2014).

Recently, we reported two different chemical approaches for large-scale release of N-glycans. The first approach is a “chemoenzymatic” method to release N-glycans called threshing and trimming (TaT) (Song et al., 2014b). In the first threshing step, glycoproteins are treated with pronase to create a pool of N-glycoamino acids and glycopeptides with short peptide moieties. In the second trimming step, N-bromosuccinimide (NBS) is added to the mixture of glycoamino acids and glycopeptides to generate free-reducing glycans, nitriles, or aldehydes, depending on different reaction conditions. These products can be easily tagged with fluorescent tags for HPLC purification, MALDI-TOF-MS analysis, and functional study. The TaT approach releases N-glycans without using specialty enzymes, hazardous chemical reagents, and harsh reaction conditions; thus, it can be easily applied for relatively large-scale glycan preparation.

Inspired by the oxidative decarboxylation by NBS treatment, we explored other oxidative reagents and surprisingly discovered that sodium hypochlorite (NaClO) (household bleach) efficiently releases glycans from most classes of natural glycoconjugates (N-glycans, O-glycans, and GSLs) directly from cells, tissues, and organs (Song et al., 2016). In this oxidative release of natural glycan (ORNG) method, household bleach is added to homogenized natural materials (animal/plant tissues) and stirred for 15–30 min at room temperature. After acid precipitation, the free-reducing N-glycans in the supernatant are purified by chromatography techniques, including size exclusion, anion exchange, and hydrophobic/hydrophilic interaction. Purified glycans are ready for fluorescent tagging by reductive amination and separated into individual components by multidimensional HPLC. The ORNG approach is fast, easy to operate, and can be applied to multi-kilograms of natural materials to produce gram-scale natural complex glycans. In our most recent study, the ORNG approach was demonstrated as a complementary route for the preparation of multi-milligram quantities of purified high-mannose N-glycans (Zhu et al., 2018a).

## O-Glycan Release From Natural Glycoproteins

Mucin-type O-GalNAc glycans, which attach to serine or threonine residues of proteins through an α-linkage, are the major O-glycans. Compared with N-glycans, which can be released from glycoproteins by several N-glycanases (Plummer et al., 1984; Tarentino et al., 1985; Plummer and Tarentino, 1991), there is a lack of effective general O-glycanase to release O-glycans. Natural O-glycans are traditionally released by chemical methods. The most commonly used method is reductive β-elimination using sodium hydroxide (NaOH) and sodium borohydride (NaBH_4_) (Carlson, 1966, 1968). Because the common 3-O-substituion at core GalNAc renders it susceptible toward a β-elimination-related peeling reaction after the release of O-glycan from the protein backbone by NaOH, *in situ* reduction of the reducing end by high-concentration NaBH_4_ is necessary. The reductive β-elimination converts the reducing end of O-glycan to alditols. Although it is useful for MS-based glycan profiling, it prevents further derivatization and functionalization for glycan purification and printing on a microarray. Several nonreductive β-elimination methods have been developed to keep the reducing end for further derivatization; (Patel et al., 1993; Chai et al., 1997; Huang et al., 2001; Merry et al., 2002; Miura et al., 2010; Yamada et al., 2010; Kozak et al., 2012) however, most of them are still based on base-catalyzed β-elimination, and “peeling” is nearly inevitable (Yu et al., 2010). Furthermore, even if an intact free-reducing end is generated, the following tagging step often generates open-ring O-glycans, which destroy the structural integrity of the O-glycans and, subsequently, may affect its functional study, such as the glycan recognition on a microarray (Prasanphanich et al., 2015). The regeneration of the natural α-O-linkage is significantly more challenging than that of the N-glycan linkage. A PMP-related releasing and tagging approach for O-glycans has also been developed by Wuhr's and Wang's groups (Wang et al., 2011; Zauner et al., 2012) using the combination of β-elimination followed by Michael addition, both of which are catalyzed by a strong base. However, the PMP or related tagged glycans are only suitable for glycomics analysis—not for further derivatization and functional screening on microarrays.

Interestingly, our novel ORNG method also can effectively release O-glycans from glycoproteins or tissues of organisms (Song et al., 2016). The release of O-glycans by ORNG is mechanistically different from all previously known methods. Instead of base-catalyzed elimination, sodium hypochlorite oxidatively degrades the protein backbone to generate O-glycan-acids containing glycolic acid (serine-linked) or lactic acid (threonine-linked) as aglycons in addition to a smaller fraction of free-reducing O-glycans. As a result, these glycolic/lactic acid–linked O-glycans to a great extent retain the structural integrities of the O-glycans as well as the α-O-linkage to the aglycon, preserving O-glycan recognition involving the linkage. In addition, compared to β-elimination, ORNG release is faster and the reaction condition is milder; thus, many labile functional groups, such as sulfation and O-acetylation, are uncompromised after NaClO treatment. More importantly, the released O-glycan acids can be easily labeled using a common amidation reaction with a florescent tag, such as mono-9-florenyl-methoxycarbonyl (mono-Fmoc) ethylenediamine for HPLC separation to prepare O-glycan libraries, and these mono-Fmoc tagged O-glycans can be deprotected by piperidine to expose the amino group for immobilization onto microarray slides for functional O-glycomics studies.

**Figure 3:**
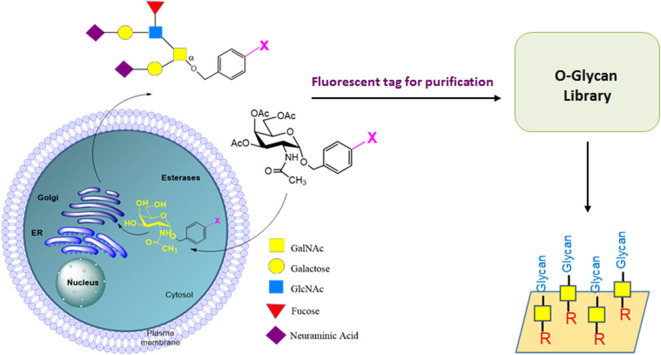
CORA method for preparation of O-glycans by living cells. Ac_3_Bn-α-GalNAc derivative can enter the cell, be deacetylated to form a Bn-α-GalNAc derivative, and then be extended by glycosyltransferases in the O-glycosylation pathway in Golgi. The Bn-O-glycan derivatives are secreted to cell media. The fluorescently labeled O-glycans can be purified to prepare O-glycan libraries for functional O-glycome study.

Unlike all the above release strategies, recently we have developed a novel technology termed cellular O-glycome reporter/amplification (CORA), which uses an O-glycan precursor (peracetylated benzyl-α-N-acetylgalactosamine, Ac_3_Bn-α-GalNAc) to amplify O-glycans in living cells and secretes free Bn-O-glycans into the cell media. The secreted Bn-O-glycans can be easily purified and analyzed by MS (Kudelka et al., 2016). CORA greatly enhances the sensitivity of MS analysis of O-glycome from living cells. However, the low UV absorption of the Bn group makes the isolation of these glycans using HPLC challenging. In order to overcome this limit, we have recently designed and synthesized many Ac_3_Bn-α-GalNAc derivatives as CORA precursors to replace Ac_3_Bn-α-GalNAc. These new CORA precursors include many function groups, such as the fluorescence group and bioorthogonal reactive groups (Zhang et al., 2019), allowing O-glycans produced by CORA to be tagged, separated, and purified by chromatography for functional study. Preparative CORA using these derivatives as precursors is currently under investigation, and we believe this method could become a promising approach for preparation of O-glycans (Figure 3).

## Glycan Release From Glycosphingolipids

Glycosphingolipids (GSLs) are amphipathic glycoconjugates widely distributed on the cell surfaces. Although exoglycosidases and endoglycosidases are only able to cleave the glycan moieties from GSLs (Li and Li, 1999), endoglycoceramidases are found to release entire glycans from GSLs (Ishibashi et al., 2007; Li et al., 2009; Albrecht et al., 2016). However, the enzymes are expensive and specific to certain GSL structures, preventing their wide application in larger scale glycan preparation from GSLs.

Traditional chemical methods utilize ozonolysis or osmium tetraoxide to oxidize the C=C double bond in the sphingosine moiety, followed by base-catalyzed β-elimination (Wiegandt and Baschang, 1965; Hakomori, 1966). In order to prevent the potential adverse effect of base treatment on glycan structural integrities, we have developed several approaches to release glycans from GSLs for functional study through glycan microarray preparation using covalent immobilization. The first approach takes advantage of the aldehyde group generated by ozone treatment of GSLs, which can be directly coupled with functional and fluorescent tags by reductive amination. This approach preserves a significant portion of the lipid moiety and may benefit functional studies requiring the lipid component (Song et al., 2011). The second approach is to heat ozonized GSLs gently under neutral pH, which interestingly releases free-reducing glycans fairly efficiently (Song et al., 2012). Both of these methods still require ozone to oxidize the C=C double bond to initiate the reaction and can only be applied to purified GSLs. In our most recent ORNG approach, we found that in addition to N- and O-glycans, NaClO can also release glycans as cyanomethyl glycosides from GSLs—apparently through the oxidative degradation of the lipid moiety at the polar head group (Song et al., 2016). The ORNG approach can be applied not only to gangliosides purified by organic solvent extraction, but also directly to aqueous homogenized brain tissue (Song et al., 2016). The ability to release GSL-glycans without involving organic solvent extraction significantly reduced the complexity of GSL-glycan preparation and is essential to larger scale glycan production. Interestingly, although NBS can also release glycan nitriles from gangliosides at 65°C, this reaction does not work directly on homogenized brain tissue.

## Glycan Release From Glycosaminoglycans and GPI-Anchors

Glycosaminoglycans (GAGs) are linear polydisperse heteropolysaccharides, consisting of up to 1,000 repetitive disaccharide units (Murata et al., 1985; Jackson et al., 1991). Heparin, a highly sulfated form of heparan sulfate (HS) glycosaminoglycans, has been shown to possess important biological functions that vary according to its fine structure (Liu et al., 2009). Heparin has widespread clinical use as an intravenous anticoagulant with more than 100,000 kg produced annually worldwide (Liu et al., 2009). Commercial heparin is currently produced from animal tissues, such as porcine intestine and beef lung (Bhaskar et al., 2012). The methods used for commercial preparation of heparin involve five basic steps: (1) preparation of tissue, (2) extraction of heparin from tissue, (3) recovery of raw heparin, (4) purification of heparin, and (5) recovery of purified heparin (Linhardt and Gunay, 1999). While being similar, the heparins derived from different animal sources have diverse structures that relate to different functional activities, such as AT- and thrombin-binding affinities (Liu et al., 2009). A worldwide health crisis in 2007, associated with contamination of several heparin batches, reportedly resulted in more than 200 deaths alone in the United States (Liu et al., 2009; Turnbull, 2011). Low-molecular weight heparins (LMWH, MW avg <8 kDa) are subcutaneously administered, have a longer half-life than unfractionated heparin, and can be prepared with different structures by different depolymerization methods, including oxidation, deaminative degradation, and β-elimination (Linhardt and Gunay, 1999).

With the improvement in chemical and chemo-enzymatic methods, the synthetic scale of GAGs has reached gram scale, and the automated solid-phase synthesis of chondroitin sulfate GAGs is available (Eller et al., 2013; Mende et al., 2016; Xu et al., 2017), which enable facilitated access to functional and biological study of GAGs. Although glycan microarray analysis of natural GAG oligomers have been reported for more than 10 years (Noti et al., 2006; Park et al., 2008), large-scale GAG microarrays for general screening of GAG-binding proteins are only reported in a synthetic approach (Yang et al., 2017; Zhang et al., 2017; Zong et al., 2017). The high heterogeneity of the sulfation patterns of the GAG chains make the isolation of homogeneous GAG oligomers, structural characterization, and chemical/enzymatic synthesis a challenging task. Nevertheless, with the recent progress in HPLC analysis and separation, preparation of a more comprehensive natural GAG glycan library for functional study with GAG-binding proteins will become possible in the near future.

GPI-anchor proteins play critical roles in numerous biological processes, such as cell recognition and interaction (He et al., 1987; Takeda and Kinoshita, 1995; Paulick and Bertozzi, 2008). Because the first total synthesis of an intact GPI anchor was in 1991 (Murakata and Ogawa, 1991), convergent chemical and chemo-enzymatic strategies for GPI synthesis were developed, and more than 30 GPIs were isolated and characterized (Wu et al., 2008; Yu and Guo, 2009; Swarts and Guo, 2010; Guo, 2013). AN effective strategy of labeling of cell-surface GPIs and GPI-anchored proteins was developed for biological studies (Lu et al., 2015). However, natural-sourced GPI anchor preparation for functional study is not well studied yet, presumably due to the lack of well-defined enzymatic and chemical release methods and low abundance of GPI-anchors in cells.

## Preparation of Human Milk Oligosaccharides

Human milk oligosaccharides (HMOs), occurring as free-reducing glycans, are the third major component of human milk after lactose and lipids and are known to play important roles benefiting infant health (Chen, 2015). HMOs are extended from lactose by a collection of glycosyltransferases, adding N-acetyl-glucosamine, galactose, fucose, and neuraminic acid (Jenness, 1979). More than a hundred different HMO structures have been identified and elucidated (Zopf et al., 1978; Prieto and Smith, 1985; Smith et al., 1985; Jensen et al., 1995). Due to its high abundance in human milk (5–15 g/L) as free-reducing glycan without the need to release from other biomolecules, large-scale isolation and separation of HMOs have been practiced for many years. In an early study, individual HMOs were isolated directly by size exclusive, anion-exchange, and paper chromatography without being derivatized (Kobata et al., 1969; Donald and Feeney, 1988). More recently, with the wide use of HPLC isolation and MS analysis, tagging of HMOs by functional and/or fluorescent groups for separation and further functional study is more common. We have applied our bifunctional fluorescent tag AEAB to HMO isolation and fractionation (Song et al., 2009b). Isolated glycans can be directly printed on a microarray for functional screening with various GBPs and viruses (Yu et al., 2012). With more complex HMO structures becoming available for functional study, we expect further elucidation of their functions through interaction with the infant microbiome.

## Functional and Fluorescent Tagging of Released Glycans

After being released from natural sources, glycans existing in the heterogeneous mixture need to be separated for analysis or for preparation of pure glycans. Due to the lack of an exploitable chromophore in natural glycans and the anomeric mutual rotation at the reducing end, it's a challenge to monitor glycans during HPLC separation. The preparation of glycan microarrays also requires that the glycans are derivatized with functional groups, such as an amino group. Therefore, it is important to install functional and fluorescent tags on the released glycans for easier and more efficient separation and solid phase immobilization afterward.

**Figure 4:**
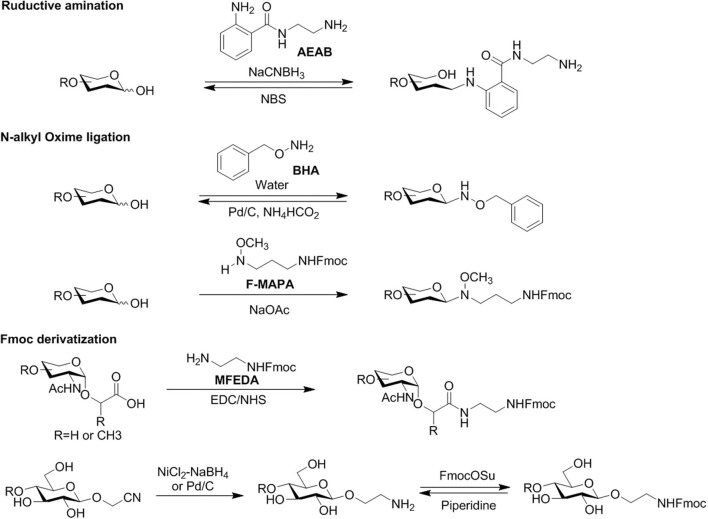
Several typical methods of fluorescent tagging of released glycans for functional study preparation.

Reductive amination of free-reducing glycans with fluorescent amines has long been used for the HPLC profiling of glycans (Figure 4). 2-aminopyridine (2-AP), 2-aminobenzamide (2-AB), 2-aminobenzoic acid, or anthranilic acid (2-AA) are commonly use fluorescent amines (Hase et al., 1978; Bigge et al., 1995; Anumula, 2014). However, these small fluorescent amines lack a functional group for efficient solid phase immobilization or covalent derivatization. With an aromatic amino group, a homobifunctional tag, 2,6-diaminopyridine (DAP) conjugated glycans can be immobilized onto activated surfaces for microarray preparation (Xia et al., 2005; Song et al., 2008). To efficiently immobilize precious natural glycans, we developed a novel heterobifunctional tag, 2-amino-N-(2-aminoethyl)benzamide (AEAB), which contains both arylamine and alkylamine (Song et al., 2009b). The aromatic amine selectively reacts with the free-reducing end of released glycans by reductive amination while the alkylamine is used for efficient solid-phase immobilization onto both NHS and epoxy-activated glass slides.

One inherent problem with commonly used reductive amination is breaking the reducing end ring structure, affecting the glycan structural integrity. To address this drawback, new methods and linkers have been reported, such as 2-amino-methyl-N,O-hydroxyethyl (AMNO) (Bohorov et al., 2006) and N-Fmoc-3-(methoxyamino)propylamine (F-MAPA) (Wei et al., 2019). We also developed a procedure to prepare HMO-AEAB conjugates with an intact reducing end ring structure (Yu et al., 2012). More recently, we have designed a new tag, O-benzylhydroxylamine (BHA), which can be easily and efficiently installed on HMOs and keep the glycan structure integrity (Zhang et al., 2020). By Pd/C-catalyzed hydrogenation, free HMO can be easily regenerated from HMO-BHA.

Compared to nonderivatized glycans, the installation of a fluorescent tag to released glycans often increases the sensitivity of MS analysis. Although premethylation is considered a necessary step for detailed sequencing by MS (Ashline et al., 2014), the conjugated tags often generate structural complexity during permethylation. Therefore, we developed a facile and mild method using NBS to remove tags of aminated glycans, which regenerates free-reducing glycans for permethylation (Song et al., 2013). This method can be efficiently applied to all types of tags installed through reductive amination, including 2-AP, 2-AB, 2-AA, and AEAB.

Because of the easy installation and removal, the 9-fluorenylmethoxycarbonyl (Fmoc) group is widely used as an amino-protecting group in organic chemistry, especially in peptide synthesis. After being installed on released glycans, the fluorescent Fmoc group can greatly enhance sensitivity of HPLC to tagged glycans (Kamoda et al., 2005; Song et al., 2009a; Yamada et al., 2013; Lu et al., 2019). It can also serve as an affinity tag due to the hydrophobicity. The amino group can be easily regenerated for solid-phase immobilization in microarray printing (Kamoda et al., 2005; Yamada et al., 2013; Wei et al., 2019). We have successfully installed an Fmoc tag on the released glycan from natural O-glycanconjugates and glycosyphingolipids in our ORNG method (Song et al., 2016).

## HPLC Separation of Glycans for Functional Study

Over the years, various HPLC methods have been commonly used for glycan purification, including hydrophilic interaction liquid chromatography (HILIC), high-performance anion-exchange chromatography (HPAEC), and reversed-phase chromatography (Ruhaak et al., 2010; Nagy et al., 2017). HILIC mode HPLC is an efficient technique for separation of unprotected saccharides (Fu et al., 2010; Melmer et al., 2011; Wan et al., 2015) while reversed-phase chromatography is suitable for hydrophobic saccharides (Rajakylä, 1986; El Rassi, 1995; Dallabernardina et al., 2016). HPAEC is usually used for negatively charged unprotected carbohydrates (Rohrer et al., 2013, 2016). Porous graphitized carbon (PGC) as a unique stationary phase combining both hydrophobic and anionic interactions separates glycans based very well on their isomeric structures under reverse-phase elution conditions (Fan et al., 1994; Itoh et al., 2002; Ruhaak et al., 2009; West et al., 2010; Lie and Pedersen, 2018). Because the glycans obtained from biological sources are often complex mixtures, multidimensional HPLC is necessary to separate them into individual glycans with significant purity (Nagy et al., 2017). We have successfully applied multidimensional HPLC to isolate an individual glycan library for microarray study (Song et al., 2009b; Yu et al., 2012).

Most of the HPLC separation methods are designed for analytical glycomics using small samples, which does not generate significant quantities of glycans for detailed functional study. There have been a few examples in which a more significant amount of starting materials are used to generate a sufficient amount glycans for NMR study (Green et al., 1988; Da Silva et al., 1995). However, no real preparative-scale HPLC separations have been tacked previously, presumably due to the unavailability of a large amount of released glycans. With gram-scale glycans from a natural source are available because of the ORNG technique, development of preparative-scale purification becomes practical and provides an effective route to address the lack of glycans for functional study. We have reported isolation of high mannose N-glycans from soy proteins and egg yolks by a preparative scale multidimensional HPLC method (Zhu et al., 2018a,b). However, even after multidimensional HPLC, some fractions are still mixtures of isomers that are very difficult to separate even on analytical columns. To address this problem, recycled HPLC could be a good solution (Alley et al., 2013; Sidana and Joshi, 2013). Most recently, we have reported a simple and affordable closed-loop recycled HPLC method for separation of complex glycans in the preparative scale. It was successfully applied to reverse-phase chromatography, HILIC, and sizes using size-exclusion chromatography (SEC) (Zhu et al., 2020).

## Conclusions

With a highly diverse structure, natural glycans are likely more biologically relevant for functional study. Here, we have summarized the preparation of several classes of complex glycans from glycoconjugates. Both enzymatic and chemical approaches have been discussed, and each method has its own advantages and should be carefully selected based on the specific goal of individual study. When a large amount of natural glycans are desired, chemical approaches, especially the new ORNG approach provides a good alternative to chemoenzymatic synthesis. The ORNG approach is able to quickly release up to grams of glycans from several major classes of glycoconjugates using affordable chemical reagents (household bleach), a mild reaction condition, and a simple operation. Nevertheless, more preparation methods are still in demand, especially for O-glycans, GAGs, and GPI-anchors. The novel CORA method provides a potential new route toward O-glycans if preparative scale can be achieved.

## Author Contributions

QZ, ZL, and XS wrote and edited the manuscript. All authors contributed to the article and approved the submitted version.

## Conflict of Interest

XS is a co-founder of NatGlycan LLC, which commercializes the ORNG process. The remaining authors declare that the research was conducted in the absence of any commercial or financial relationships that could be construed as a potential conflict of interest.
